# Optimization of the Ethanol Recycling Reflux Extraction Process for Saponins Using a Design Space Approach

**DOI:** 10.1371/journal.pone.0114300

**Published:** 2014-12-03

**Authors:** Xingchu Gong, Ying Zhang, Jianyang Pan, Haibin Qu

**Affiliations:** Pharmaceutical Informatics Institute, College of Pharmaceutical Sciences, Zhejiang University, Hangzhou, 310058, China; University of Nebraska Medical Center, United States of America

## Abstract

A solvent recycling reflux extraction process for *Panax notoginseng* was optimized using a design space approach to improve the batch-to-batch consistency of the extract. Saponin yields, total saponin purity, and pigment yield were defined as the process critical quality attributes (CQAs). Ethanol content, extraction time, and the ratio of the recycling ethanol flow rate and initial solvent volume in the extraction tank (RES) were identified as the critical process parameters (CPPs) via quantitative risk assessment. Box-Behnken design experiments were performed. Quadratic models between CPPs and process CQAs were developed, with determination coefficients higher than 0.88. As the ethanol concentration decreases, saponin yields first increase and then decrease. A longer extraction time leads to higher yields of the ginsenosides Rb_1_ and Rd. The total saponin purity increases as the ethanol concentration increases. The pigment yield increases as the ethanol concentration decreases or extraction time increases. The design space was calculated using a Monte-Carlo simulation method with an acceptable probability of 0.90. Normal operation ranges to attain process CQA criteria with a probability of more than 0.914 are recommended as follows: ethanol content of 79–82%, extraction time of 6.1–7.1 h, and RES of 0.039–0.040 min^−1^. Most of the results of the verification experiments agreed well with the predictions. The verification experiment results showed that the selection of proper operating ethanol content, extraction time, and RES within the design space can ensure that the CQA criteria are met.

## Introduction

Solvent recycling reflux extraction is an extraction process wherein extraction and concentration are conducted simultaneously [1]. The extract in the extraction tank is pumped out and concentrated in the concentration tank during the extraction process; meanwhile, the evaporated solvent is condensed and pumped back into the extraction tank. Compared with the conventional heat reflux extraction, solvent recycling reflux extraction has several advantages [2]. Because the solvent is renewed in the extraction, the mass transfer driving force is greater, which leads to a shorter extraction time. The reuse of the solvent in the extraction also decreases the amount of solvent needed. In addition, no storage tank is required before the concentration process, which can reduce the fixed investment. The high efficiency of the solvent recycling reflux extraction process was verified in the extraction of polysaccharides from *Grifola frondosa* [3] and the preparation of Jianwei Xiaozhang pills [4], Xinmaikang tablets [1], and other botanical extracts [2]. Because of these advantages, solvent recycling reflux extraction is increasingly applied to extract botanical components in botanical medicine factories to lower costs.

In addition to economic considerations, botanical drug quality is important for botanical drug manufacturers. Because of the complexity of the compositions of botanical extracts, maintaining batch-to-batch consistency is a challenging task. Quality by design (QbD) is a paradigm that has recently been used to improve the batch-to-batch consistency of the pharmaceutical process based on risk management and knowledge management [5], [6]. In the implementation of the QbD concept, steps including critical quality attribute (CQA) definition, risk assessment, critical process parameter (CPP) determination, design space development, control strategy design, and continual improvement in the product lifecycle are required [7], [8]. The design space is a region for the control of process parameters. When the parameters vary within this region, the change in the process product quality can be neglected [7]. To determine the design space, mathematical models between CQAs and CPPs are required. Experimental design is often applied to establish the models [9]. To quantify the ability of the design space to keep the CQAs within the desired ranges, the probability of attaining the CQA ranges must be calculated [10]. Monte-Carlo and Bayesian methods are commonly used to calculate this probability [11]–[13]. Recently, ethanol precipitation and water precipitation, two separation processes that are widely applied in the manufacturing of botanical drugs [14], [15], have been successfully optimized according to the QbD paradigm.

To realize a solvent recycling reflux extraction process with high batch-to-batch consistency, the extraction of *Panax notoginseng* was investigated. *Panax notoginseng*, the root of *Panax notoginseng* (Burk.) F. H. Chen, is a medicinal and edible plant in China and is used as a dietary supplement in the USA [16]. Many botanical drugs widely applied in China are made from *Panax notoginseng*, such as Xuesaitong injections, Compound Danshen Dripping Pills, and Yunnan Baiyao. In this work, the extraction process was optimized using a design space approach [17] consisting of CQA definition, risk assessment, CPP determination, design space development and verification. The CQAs of the extraction process were defined, and the CPPs were identified via risk assessment. Quantitative models were developed between CQAs and CPPs. The influences of different parameters were discussed. The probability-based design space was calculated using a Monte-Carlo method. Finally, the design space was verified.

## Methods and Materials

### Materials and chemicals

*Panax notoginseng* was collected from Wenshan of Yunnan Province (China). No specific permissions were required for the described field studies. The locations are neither privately owned nor protected by the Chinese government. No endangered or protected species were sampled. The specific location of this study is longitude: 120.07E, latitude: 30.28N. Standards of the notoginsenoside R_1_, ginsenoside Rg_1_, ginsenoside Rb_1_, and ginsenoside Rd were purchased from Shanghai Winherb Pharmaceutical Technology Development Co., Ltd. (Shanghai, China). Acetonitrile (HPLC grade) and methanol (HPLC grade) were obtained from Merck (Darmstadt, Germany). Formic acid (HPLC grade) was purchased from Tedia (Darmstadt, Germany). Ethanol (analytical grade) was purchased from Shanghai Lingfeng Chemical Reagent Co., Ltd. (Shanghai, China). Tartrazine (HPLC grade) was purchased from Aladdin Industrial Corporation (Shanghai, China). Glycerol (analytical grade) was purchased from China Sun Specialty Products Co., Ltd. (Changshu, China). A Milli-Q academic water purification system (Milford, MA, USA) was used to produce deionized water.

### Procedures

**Figure 1:**
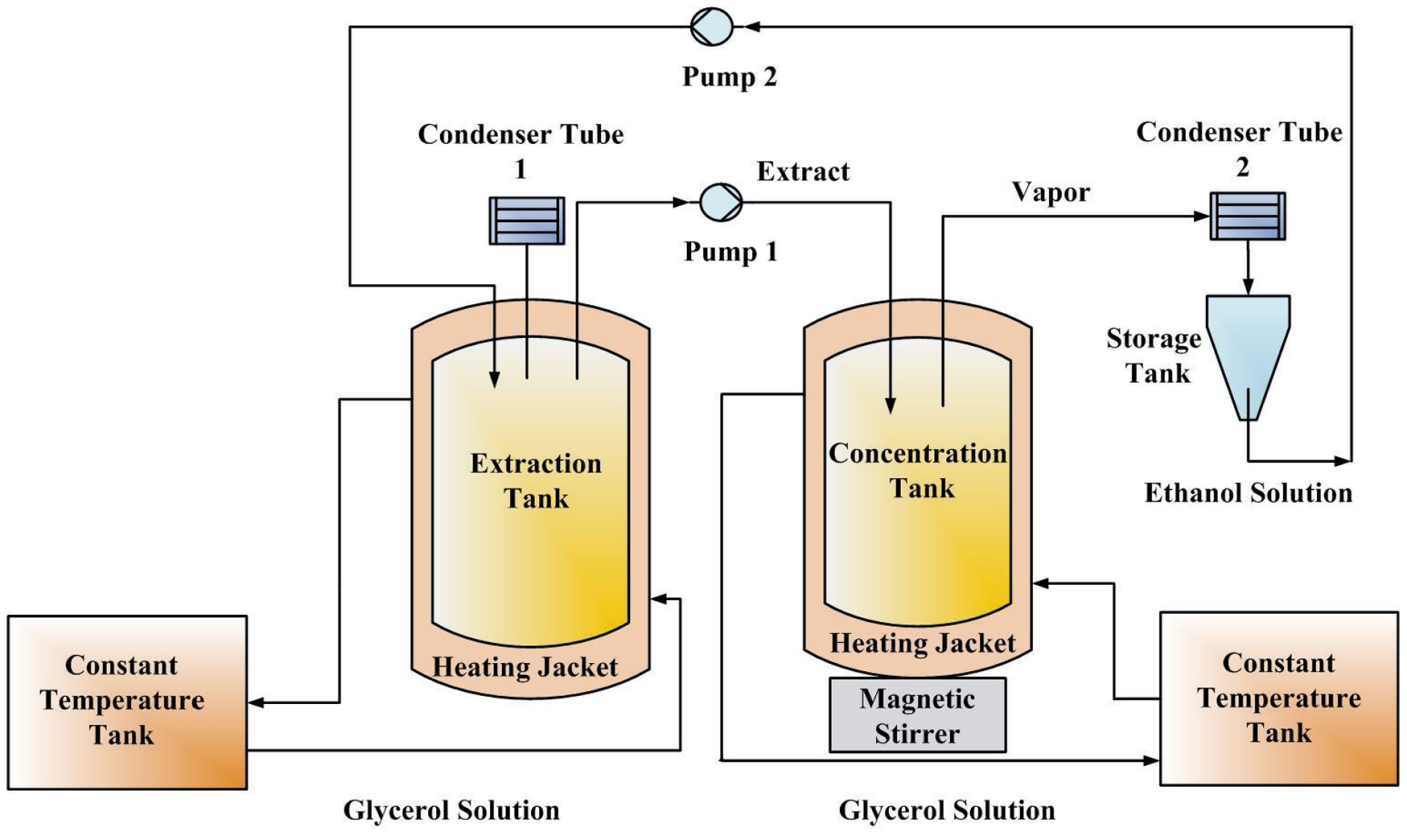
Schematic chart of the experimental setup.

A schematic chart of the experimental setup is shown in Figure 1. Two constant-temperature tanks (ZCY-15B, Ningbo Tianheng Instrument Factory, Ningbo, China) were used to heat the extraction tank and concentration tank. The extract in the extraction tank was pumped into the concentration tank. The solvent was evaporated in the concentration tank and then condensed. The ethanol solution was collected in a storage tank and then pumped back into the extraction tank with a fixed flow rate. In this process, the saponins were kept in the concentration tank, and the solvent was recycled. In the experiments, 50.0 g of *Panax notoginseng* and 500.0 ml of ethanol-water mixture were added to the extraction tank. After soaking for 2 h, 150 mL of extract was pumped into the concentration tank at a flow rate of 25 mL/min. The flow rates of the two pumps were set to the value required by the experimental design.

### Experimental design

The influences of three independent variables, namely, the ethanol concentration (v/v, X_1_), extraction time (h, X_2_), and ratio of recycling ethanol flow rate and initial solvent volume in the extraction tank (RES, min^−1^, X_3_), were investigated. A three-variable, three-level Box–Behnken design (BBD) was employed. The coded and uncoded values of the three independent variables are given in Table 1. The run order of the experiments is listed in Table 2. After the design space was developed, verification experiments with the conditions listed in Table 3 were carried out and repeated three times.

**Table 1:** Coded and uncoded values of factors.

Parameters	Symbols	Coded values		
		−1	0	1
Ethanol concentration (v/v, %)	X_1_	70	80	90
Extraction time (h)	X_2_	4	7	10
RES (min^−1^)	X_3_	0	0.02	0.04

**Table 2:** Box-Behnken design experiments and results.

Run order	X_1_	X_2_	X_3_	Saponin yield(mg/g *Panax notoginseng*)				TSP (%)	Pigment yield (mg/g *Panax notoginseng*)
				R_1_	Rg_1_	Rb_1_	Rd		
1	70	7	0.04	13.56	43.96	45.06	10.16	25.34	2.64
2	70	7	0	11.39	42.01	41.10	8.65	29.89	0.39
3	90	7	0	10.14	33.17	30.42	6.84	29.78	0.14
4	70	10	0.02	11.19	38.24	42.69	9.24	23.11	7.87
5	80	4	0	11.04	38.18	35.93	7.99	28.96	0.23
6	80	4	0.04	12.39	44.03	43.04	9.53	27.29	1.81
7	80	7	0.02	13.26	46.34	44.41	9.54	27.34	2.92
8	80	7	0.02	13.27	45.60	45.39	9.40	27.23	2.88
9	80	10	0.04	14.91	48.14	50.71	10.45	27.25	3.68
10	90	4	0.02	10.81	35.58	33.42	7.65	25.68	0.75
11	90	10	0.02	11.15	37.46	35.17	7.72	25.89	3.02
12	80	10	0	11.47	43.46	43.09	9.53	32.55	0.35
13	80	7	0.02	14.40	47.00	47.75	9.67	27.61	3.41
14	90	7	0.04	10.82	37.79	36.27	8.02	32.03	1.13
15	70	4	0.02	11.80	42.99	43.52	8.97	25.11	4.86
16	80	7	0.02	13.77	46.11	47.36	9.28	27.32	3.11

**Table 3:** Conditions and results of verification experiments.

Verification experiment No.		V_1_	V_2_
Ethanol concentration (%)		80	90
Extraction time (h)		7	4
RES (min^−1^)		0.04	0.04
Probability		0.97	0
Within design space		Yes	No
Notoginsenoside R_1_ yield (mg/g *Panax notoginseng*)	EV	14.04±0.20	11.01±0.32
	PV	14.14	9.60
	RD (%)	0.73	12.76
Ginsenoside Rg_1_ yield (mg/g *Panax notoginseng*)	EV	46.45±0.77	36.54±1.16
	PV	47.33	35.22
	RD (%)	1.89	3.61
Ginsenoside Rb_1_ yield(mg/g *Panax notoginseng*)	EV	47.23±0.17	33.77±0.82
	PV	47.53	33.10
	RD (%)	0.64	1.98
Ginsenoside Rd yield(mg/g *Panax notoginseng*)	EV	9.94±0.17	7.53±0.14
	PV	10.08	7.92
	RD (%)	1.40	5.22
TSP (%)	EV	28.68±0.26	30.57±0.53
	PV	29.19	29.84
	RD (%)	1.79	2.40
Pigment yield(mg/g *Panax notoginseng*)	EV	1.85±0.20	0.57±0.05
	PV	2.12	0.62
	RD (%)	14.57	8.40

EV: Experimental value

PV: Predicted value.

### Analytical methods

**Figure 2:**
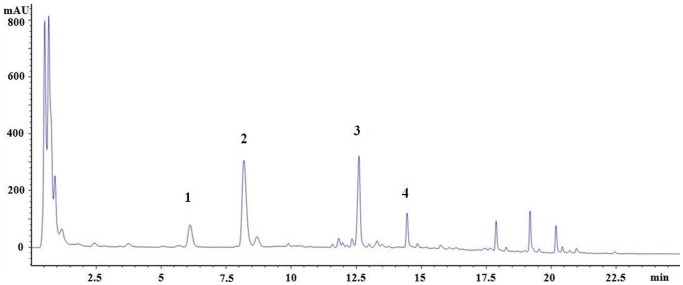
Chromatogram of Panax notoginseng extract.

The quantitative analysis of four saponins, namely, the notoginsenoside R_1_, ginsenoside Rg_1_, ginsenoside Rb_1_, and ginsenoside Rd, was performed using HPLC. HPLC analysis was performed on an Agilent 1260 series HPLC system with an Acquity UPLC CSH C18 column (50 mm×2.1 mm i.d, 1.7 µm). The column temperature was maintained at 40°C to keep column pressure in an acceptable range. The standards and samples were separated using a gradient mobile phase consisting of phase A (0.01% formic acid in deionized water) and phase B (0.01% formic acid in acetonitrile). The gradient conditions are as follows: 0–6.0 min, 18–20% B; 6.0–6.8 min, 20–30% B; 6.8–11.0 min, 30–35% B; 11.0–17.0 min, 35–90% B; and 17.0–25.0 min, 90% B. The column was then conditioned with 18% B for 15 min. The flow rate was set at 0.35 ml/min. The injection volume was 5 µL. The detection wavelength was set at 203 nm. The chromatogram is shown in Figure 2. The dry matter content was determined gravimetrically using a precision electronic balance (AB204-N, Mettler Toledo Shanghai Co., Ltd.). Before weighing, the samples were dried at 105°C in an oven (DZF-6050, Shanghai Jing Hong Laboratory Instrument Co., Ltd.) for 3 h and then stored in a desiccator for 0.5 h. The pigment content was determined using spectrophotometry. The absorbance of each sample was measured at 420 nm using a UV-vis spectrophotometer (T6, Pukinje Co., Ltd., Beijing, China). The pigment yield was calculated using tartrazine as the standard.

### Data processing

The yield of a saponin (SY) was calculated using Equation 1.
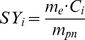
(1)where C and m refer to the extract concentration and mass, respectively; subscript i (i = 1 to 4) corresponds to notoginsenoside R_1_, ginsenoside Rg_1_, ginsenoside Rb_1_, or ginsenoside Rd, respectively; and subscripts e and pn refer to the extract and *Panax notoginseng*, respectively. The concentration of total saponin (TS) in an extract was calculated using Equation 2. 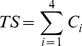
(2)


The purity of total saponin (TSP) was calculated using Equation 3.
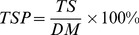
(3)where DM is the dry matter content of an extract. The yield of pigment (PY) was calculated using Equation 4.
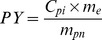
(4)where the subscript pi represents the pigment content using tartrazine as the standard.

Equation 5 was used to model the results of the Box-Behnken design experiments.
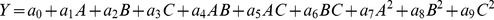
(5)where Y is the response, a_0_ is a constant, and a_1_ to a_9_ are regression coefficients. Logarithmic values were used in the model development for pigment yield. Design Expert V8.0.6.1 (State-Ease Inc., MN) was used to analyze the results of the Box-Behnken design experiments.

A self-written Matlab (R2010b, Version 7.11, MathWorks, USA) program was used to calculate the design space using a Monte-Carlo method. In the Monte-Carlo simulation, it is hypothesized that the relative standard deviations (RSD) of concentrations for all of the experimental results were the same as the RSD values of the center point. In each simulation, random data following a normal distribution were created. The simulation was carried out 50000 times to calculate the probability. The acceptable level of probability for the design space was set as 0.90.

## Results and Discussion

### Process CQA definition

Saponins are the main bioactive components of *Panax notoginseng* [18]. Saponins possess many pharmacological activities, such as antithrombotic, anti-atherosclerotic, fibrinolytic, antioxidant and cardioprotective activities [19]–[21]. Therefore, saponins are usually used as the main indices for *Panax notoginseng* product evaluation [22], [23]. Ginsenoside Rg_1_, ginsenoside Rb_1_, ginsenoside Rd, and notoginsenoside R_1_ are the *Panax notoginseng* saponins present in the highest levels [24]. Recently, the action mechanisms of ginsenoside Rg_1_, ginsenoside Rb_1_, ginsenoside Rd, and notoginsenoside R_1_ have been found to involve multiple targets and multiple pathways using a network-based approach [25]. Therefore, the yields of notoginsenoside R_1_, ginsenoside Rg_1_, ginsenoside Rb_1_, and ginsenoside Rd are selected as process CQAs. Higher saponin yields are favored. Considering that the active compound purity represents the difficulties in process quality control [26], total saponin purity is considered as a process CQA. In the extraction process, polysaccharides, pigments, salts, and other compounds will also be extracted, which will result in an increase in the dry matter yield. The dry matter yield is a function of saponin yield and total saponin purity, as seen in Equation 6. 
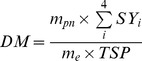
(6)


Therefore, the dry matter yield is not defined as a CQA. Color is another index of drug quality; therefore, pigment yield is also selected as a process CQA. A total of six process CQAs were taken into consideration, including the four saponin yields, total saponins purity, and pigment yield. The upper and lower limits of CQAs are given in Table 4.

**Table 4:** Upper and lower limits of the desirable ranges of CQAs.

CQA	Desirable Range	
	Low	High
R_1_ yield (mg/g *Panax notoginseng*)	13.0	15.0
Rg_1_ yield (mg/g *Panax notoginseng*)	42.0	48.0
Rb_1_ yield (mg/g *Panax notoginseng*)	43.0	51.0
Rd yield (mg/g *Panax notoginseng*)	9.0	11.0
TSP (%)	27.0	33.0
Pigment yield (equivalent of tartrazine, mg/g *Panax notoginseng*)	0.15	2.98

### CPP identification

**Figure 3:**
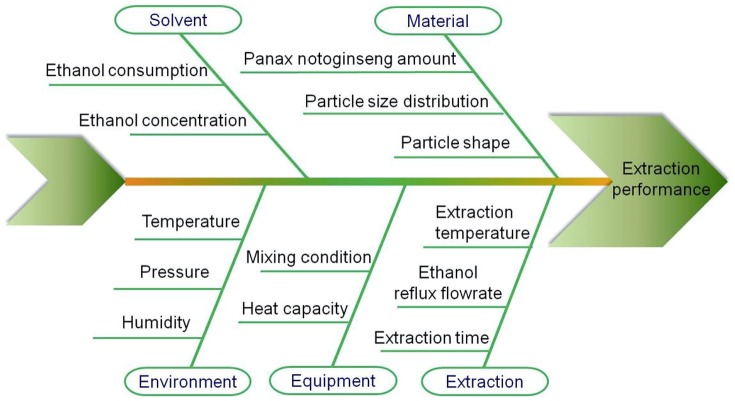
Ishikawa diagram analysis for the ethanol recycling extraction process.

Possible critical process parameters in the ethanol recycling reflux extraction process were identified using an Ishikawa diagram analysis, as shown in Figure 3. The main causes of environment, material attributes, solvent, equipment, and extraction procedure and the related sub-causes were considered.

For the further selection of critical process parameters (CPPs), a failure mode and effects analysis (FMEA) was conducted. In this analysis, a severity ranking from 1 to 4 is used to reflect the impact of each parameter on the process. The occurrence probability of a failure was ranked from 1 to 2 in each parameter. The ability to detect a failure was also ranked from 1 to 2 in each parameter. The scores for severity, occurrence, and detectability are obtained based on literature results and experience and are presented in Table 5. In Bai et al.'s work, the effect of the size distribution of *Panax notoginseng* on saponin extraction was small [27]. Instead, ethanol content, ethanol addition amount, and number of extractions have been found to be significant factors [24], [28]. The risk priority number (RPN) score was used as the criterion to identify the CPPs. The RPN was obtained by multiplying the scores for severity, occurrence, and detectability, as shown in Table 5. The parameters with an RPN score of 8 or above were selected for this study. To facilitate the transfer from the laboratory scale to a larger scale, the ratio of the recycling ethanol flow rate and initial solvent volume in the extraction tank (RES) were used to represent the effects of ethanol reflux flow rate. Therefore, the ethanol content, extraction time, and RES were identified as the CPPs.

**Table 5:** Risk assessment using FMEA for the factors of ethanol recycling extraction process.

Cause	Factors	Severity^a^	Occurrence^b^	Detectability^c^	RPN
Material	*Panax notoginseng* amount	3	1	1	3
	Particle size distribution	1	2	1	2
	Particle shape	1	1	2	2
Extraction	Extraction Temperature	3	1	1	3
	Extraction Time	4	2	1	8
	Reflux rate of ethanol	3	2	2	12
Solvent	Ethanol consumption	4	1	1	4
	Ethanol concentration	4	2	2	16
Environment	Temperature	1	1	1	1
	Pressure	1	1	1	1
	Humidity	1	1	1	1
Equipment	Mix condition	2	1	1	2
	Heat capacity	2	2	1	4

a Severity: 1, no impact; 2, small impact; 3, moderate impact; 4, remarkable impact.

b Occurrence: 1, seldom occur; 2, sometimes occur.

c Detection: 1, can be detected easily; 2, can be detected with difficulty.

### Effects of CPPs on process CQAs

The results of the Box-Behnken design experiments are shown in Table 2. The extraction yield of ginsenoside Rg_1_ varied from 33.17 to 48.14 mg/g *Panax notoginseng*. The ginsenoside Rb_1_ yield ranged from 30.42 to 50.71 mg/g *Panax notoginseng*. The yields of notoginsenoside R_1_ and ginsenoside Rd were lower than 15 mg/g *Panax notoginseng*. The TSP varied from 23.11% to 32.55%. The pigment yield was between 0.14 and 7.87 mg/g *Panax notoginseng*.

Mathematical models were developed to describe the relationships between CQAs and CPPs. The estimated regression coefficients are listed in Table 6. Analysis of variance (ANOVA) was carried out, and the p-values of the parameters are listed in Table 6. For all of the models, the determination coefficients (R^2^) are higher than 0.88, which means that most of the variations of the process CQAs can be explained by ethanol concentration, RES, and extraction time. The models are significant, as the p-values are less than 0.05. The linear term or quadratic term of ethanol concentration is important in all the models. The RES is also important for all of the criteria. Extraction time is significant for Rd yield and pigment yield.

**Table 6:** Model fitting results and ANOVA results.

Model terms	Saponins yield (mg/g *Panax notoginseng*)								TSP (%)		Pigment yield (ln(µg/g *Panax notoginseng*))	
	R_1_		Rg_1_		Rb_1_		Rd					
	Estimate	Prob>|t|	Estimate	Prob>|t|	Estimate	Prob>|t|	Estimate	Prob>|t|	Estimate	Prob>|t|	Estimate	Prob>|t|
Constant	13.6768	——	46.2633	——	46.2281	——	9.4700	——	27.3738	——	8.0307	——
X_1_	−0.6278	0.0651	−2.9012	0.0087**	−4.6364	0.0016**	−0.8498	0.0006**	1.2403	0.0211*	−0.5855	0.0002**
X_2_	0.3367	0.2723	0.8127	0.3251	1.9695	0.0612	0.3506	0.0360*	0.2196	0.6027	0.3729	0.0017**
X_3_	0.9555	0.0140*	2.1364	0.0305*	3.0673	0.0116*	0.6434	0.0026**	−1.1593	0.0274*	1.0467	<0.0001**
X_1_ X_2_	0.2365	0.5702	1.6575	0.1732	0.6454	0.6134	−0.0487	0.8002	0.5534	0.3655	0.2267	0.0604
X_1_ X_3_	−0.3715	0.3821	0.6669	0.5569	0.4740	0.7092	−0.0805	0.6777	1.6996	0.0238*	0.0399	0.6985
X_2_ X_3_	0.5233	0.2324	−0.2902	0.7958	0.1268	0.9201	−0.1566	0.4280	−0.9062	0.1601	0.0765	0.4653
X_1_^2^	−1.7090	0.0049**	−5.9570	0.0014**	−6.2568	0.0021**	−1.0188	0.0015**	−1.0889	0.1024	−0.1484	0.1816
X_2_^2^	−0.7323	0.1124	−1.7398	0.1559	−1.2746	0.3334	−0.0591	0.7594	−1.3371	0.0559	0.1414	0.2000
X_3_^2^	−0.4902	0.2598	−1.0717	0.3563	−1.7609	0.1964	−0.0353	0.8544	2.9760	0.0019**	−1.4191	<0.0001**
Model P value	0.0315*		0.0151*		0.0081**		0.0036**		0.0115*		<0.0001**	
R^2^	0.8827		0.9105		0.9283		0.9463		0.9188		0.9891	

*p<0.05

**p<0.01.

**Figure 4:**
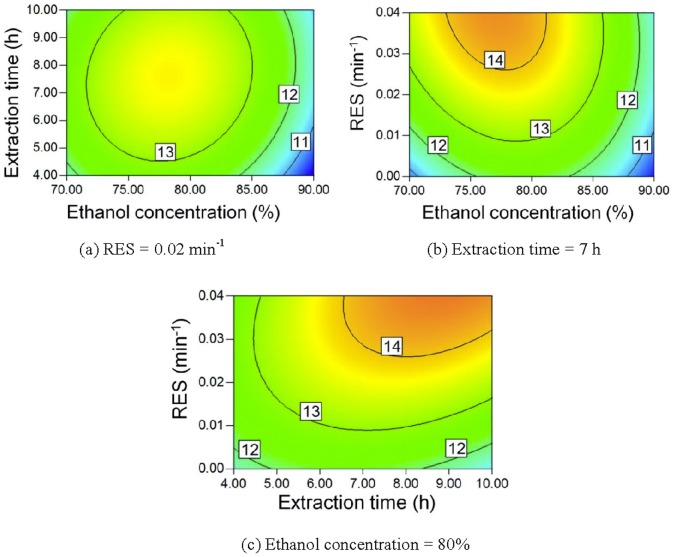
Contour plot of R_1_ yield.

**Figure 5:**
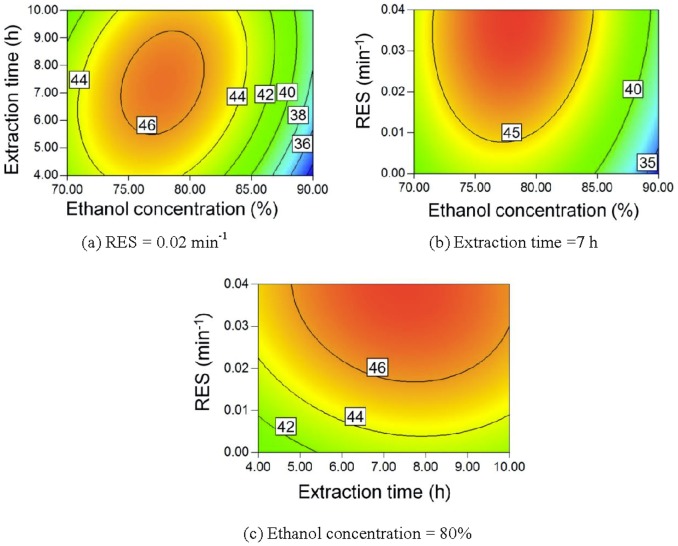
Contour plot of Rg_1_ yield.

**Figure 6:**
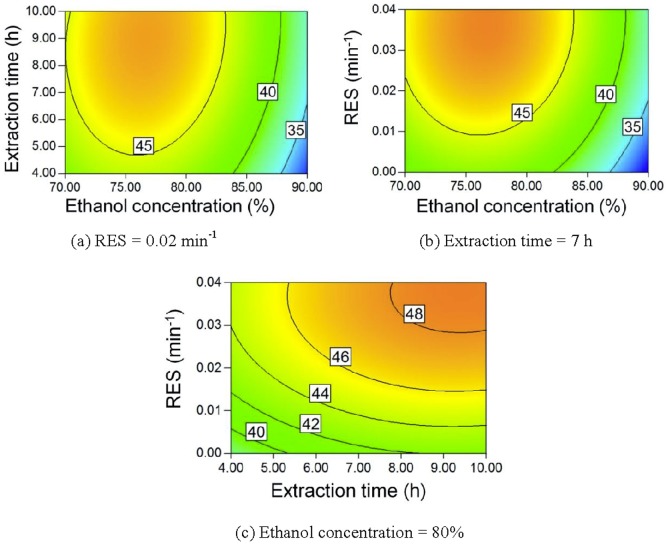
Contour plot of Rb_1_ yield.

**Figure 7:**
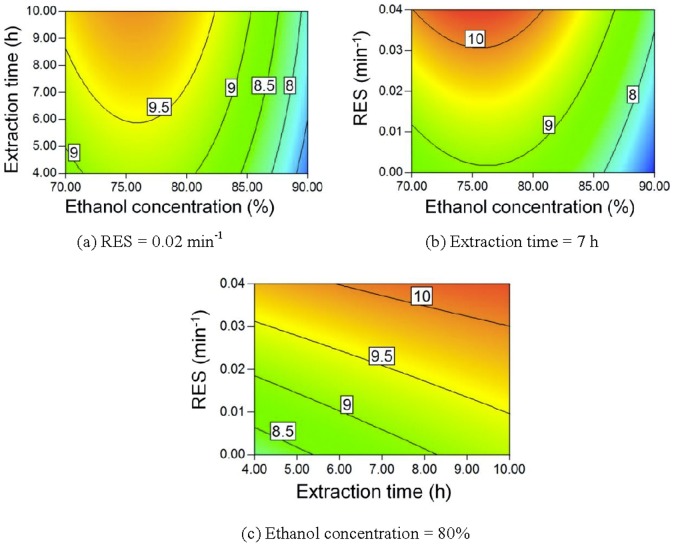
Contour plot of Rd yield.

According to the models, contour plots for saponin yields can be obtained, as given in Figures 4–7. As the ethanol concentration increases, the yields of all of the saponins first increase and then decrease. A lower ethanol content means more water in the extract, which can result in increased swelling of *Panax notoginseng*. Saponins can be extracted in a shorter time when the swelling of *Panax notoginseng* is greater. However, more saponins may hydrolyze when the water content in the mixed solvent is higher because of the higher boiling temperature [16], [18], [29], [30]. More ginsenoside Rb_1_ and ginsenoside Rd can be extracted using longer extraction times. However, the yields of notoginsenoside R_1_ and ginsenoside Rg_1_ may decrease for very long extraction times. Ginsenoside Rg_1_ is the hydrolysis product of notoginsenoside R_1_ [16]. The hydrolyzation of ginsenoside Rg_1_ forms ginsenoside Rh_1_ [16]. A higher RES leads to higher concentration differences between *Panax notoginseng* and the extract for a saponin, which accelerates the saponin extraction. Therefore, the saponin yields increase as the RES increases.

**Figure 8:**
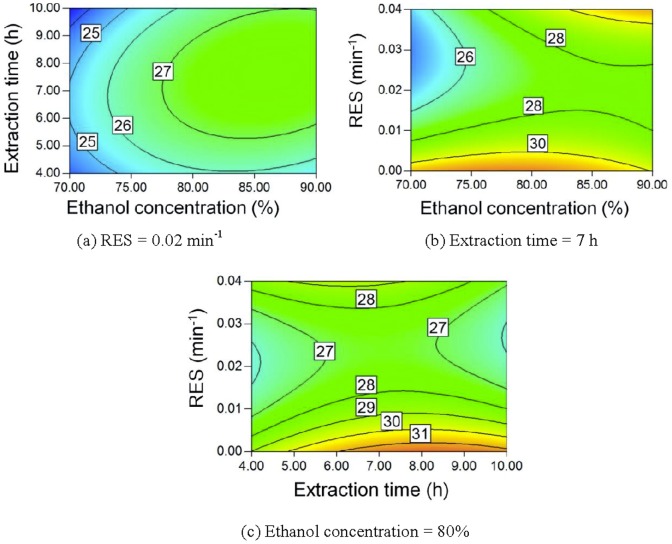
Contour plot of TSP.

The mass of saccharides, such as sucrose, fructose, and sucrose [31], was more than 60% of the dry matter content in the water extract of *Panax notoginseng* [32]. Saccharide solubilities usually decrease as the ethanol content in the mixed ethanol-water mixture increases [33]–[36]. Therefore, a higher ethanol concentration means that less saccharides will be extracted and a higher TSP can be obtained, as seen in Figure 8. The extract is easily saturated by saccharides, but the recycling of the ethanol solution can prevent this saturation, thereby allowing more saccharides to be extracted. Accordingly, a lower RES also results in a higher TSP.

**Figure 9:**
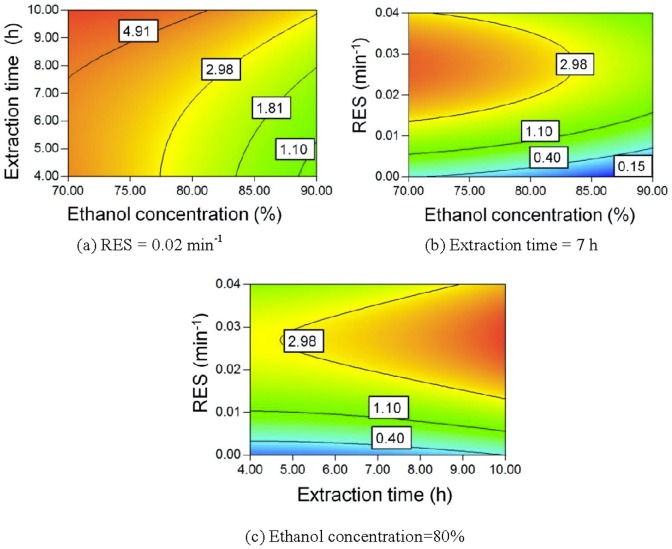
Contour plot of pigment yield.

Maillard reaction between reducing sugars and amino acids forms aroma compounds, ultra-violet-absorbing intermediates, and melanoidins, which results in a darkened extract [37], [38]. Longer extraction times result in a larger amount of Maillard reaction products. Accordingly, the pigment yield increases, as seen in Figure 9(a) and 9(c). A lower ethanol concentration corresponds to a higher water content in the extract and a higher extraction temperature for reflux. Water is also a reactant in the Maillard reaction [39]. Therefore, a lower ethanol concentration also leads to a higher pigment yield, as seen in Figure 9(a) and 9(b).

### Design space development and verification

**Figure 10:**
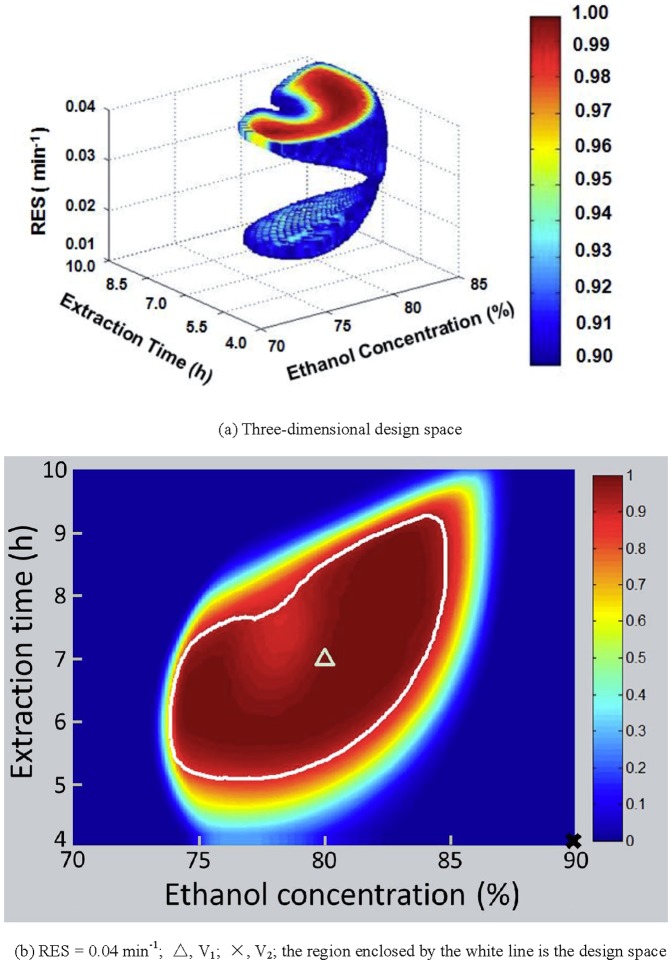
Design space and verification experiments.

The design space with a possibility above 0.90 of satisfying the CQA criteria is calculated using a Monte-Carlo method. The results are shown in Figure 10(a). The design space is irregular in shape and composed of two parts. For easier operation, normal operation ranges are calculated, comprised of an ethanol concentration of 79–82%, extraction time of 6.1–7.1 h, and RES of 0.039-0.040 min^−1^. The probability of satisfying the CQA criteria is 0.914 when the parameters are controlled within the normal operation region.

To further confirm the accuracy of the models as well as the design space, verification experiments were carried out. The experimental conditions and results are shown in Table 3. The conditions of Experiments V_1_ and V_2_ are plotted in Figure 10(b). The relative deviation (RD) values between the experimental and predicted values are calculated using Equation 7.
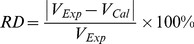
(7)where V_Exp_ is the average value of the verification experimental data, and V_Cal_ is the calculated value of the model. The RD values are less than 6% for ginsenoside Rg_1_ yield, ginsenoside Rb_1_ yield, ginsenoside Rd yield, and TSP of the extract, indicating that these models are accurate. The results of Experiment V_1_ are within the target ranges of the CQAs. However, the yields of the four different saponins in Experiment V_2_ are lower than their criteria. Thus, operation within the design space can ensure that all CQAs are within the predefined limits.

## Conclusion

The design space approach is applied to optimize the solvent recycling reflux extraction process of *Panax notoginseng*. The notoginsenoside R_1_ yield, ginsenoside Rg_1_ yield, ginsenoside Rb_1_ yield, ginsenoside Rd yield, pigment yield, and total saponin purity in the extracts were defined as the process CQAs. An Ishikawa diagram was applied to identify potential CPPs. Ethanol concentration, extraction time, and RES were identified as CPPs using an FMEA model. The models between the CPPs and process CQAs were built using quadratic models with R^2^ values greater than 0.88. As the ethanol concentration increases, the saponin yields first increase and then decrease. For the ginsenosides Rb_1_ and Rd, a longer extraction time leads to higher yields. The total saponin purity increases as the ethanol concentration increases. Meanwhile, the pigment yield decreases with increasing ethanol concentration or decreasing extraction time. The design space was calculated via Monte-Carlo simulation using 0.90 as the acceptable probability. Normal operation ranges were also recommended, namely, an ethanol concentration of 79–82%, extraction time of 6.1–7.1 h, and RES of 0.039–0.040 min^−1^. The probability of satisfying the CQA criteria with parameters in normal operation ranges is higher than 91.4%. The design space was verified, and the results of the verification experiment showed that the use of operating CPPs within the design space provides a high probability of satisfying the process CQA criteria.
